# Medialization of trochlear groove was correlated with extended lateral trochlear in trochlear dysplasia: a transverse CT analysis

**DOI:** 10.1186/s13018-022-03166-6

**Published:** 2022-05-15

**Authors:** Conglei Dong, Chao Zhao, Lingce Kong, Kang Piao, Kuo Hao, Fei Wang

**Affiliations:** Department of Orthopaedic Surgery, Third Hospital of Hebei Medical University, Ziqiang Road 139, Shijiazhuang, 050051 Hebei China

**Keywords:** Knee, Recurrent patellar instability, Trochlear dysplasia, Trochlear width, Trochlear orientation

## Abstract

**Purpose:**

To investigate the difference of trochlear width between normal and dysplastic trochlear and to analyze whether the medialization of trochlear groove was correlated with abnormal width of trochlear facets in trochlear dysplasia.

**Methods:**

This study involved CT scans of fifty knees with trochlear dysplasia (TD group) and fifty knees without obvious trochlear dysplasia (Normal group). The linear distance from the medial femoral epicondyle to the various reference points was measured on axial CT images which included the medial edge of medial trochlear facet (*d*MTE), trochlear groove (*d*TG), and the lateral edge of lateral trochlear facets (*d*LTE). The medial and lateral trochlear width was calculated and standardized by the width of the anatomical epicondylar axis. Pearson’s correlation analysis was performed between the *d*TG and the width of the medial and lateral trochlear.

**Results:**

The reliability of the results was good. Intraclass correlation coefficient (ICC) ranged from 0.89 to 0.97. The *d*MTE was significantly greater in the TD group than the normal group (32.7 ± 5.1% vs. 29.6 ± 3.5%, *p* = 0.009). There was no significant difference in the *d*LTE between groups. The *d*TG was reduced in the TD group compared with the normal group (45.2 ± 4.1% vs. 49.1 ± 3.9%, *p* = 0.019). In the TD group, there was a significant reduction in the medial trochlear width (13.9 ± 4.1% vs. 19.4 ± 2.9%, *p* < 0.001) and a significant increase in the lateral trochlear width (31.3 ± 4.0% vs. 26.9 ± 3.6%, *p* < 0.001) compared with the normal group. The *d*TG was significantly correlated with the lateral trochlear width (*r* value = − 0.693, *p* < 0.001) and not correlated with the medial trochlear width (*r* value = 0.044, *p* = 0.766) in trochlear dysplasia.

**Conclusions:**

This study demonstrated that dysplasia of trochlear morphology was related to the reduction of medial trochlear width and increase in lateral trochlear width. The medialization of trochlear groove was significantly correlated with the increased lateral trochlear width.

## Introduction

The femoral trochlear was known as one of the important factors influencing patellofemoral stability, and the dysplasia of femoral trochlear was found exists in most of patients with recurrent patellar instability [1–3]. Trochlear groove with appropriate groove depth and inclined trochlear slopes provided an osseous trajectory for patellar tracking and prevent the lateral transition of the patellar when the knee flexed over thirty degrees [2, 4]. It had been reported that contact area and pressure in the patellofemoral joint were significantly altered by the dysplastic trochlear due to the pathological shallow sulcus and irregular trochlear facets [5]. Methods on quantifying the abnormalities of trochlear dysplasia were diversiform in current studies [2, 6, 7]. On axial images, trochlear with a sulcus angle greater than145° was considered a shallow trochlear sulcus [7, 8]. It was found that a lateral trochlear inclination of fewer than 11 degrees was an effective way in distinguishing trochlear dysplasia [9, 10]. In individuals, the geometry of trochlear dysplasia had large variations and was difficult to be fully evaluated by angular measurements when the trochlear groove turned flat or convex [11]. The linear measurements measuring the sulcus depth and trochlear height were reported also reliable in quantifying trochlear dysplasia [12, 13]. The trochlear groove was not always symmetrical and neutral oriented in dysplastic trochlea [14, 15]. Studies had shown that the distance between the trochlear center and posterior cruciate ligament insertion was decreased on axial images and indicated the trochlear groove was oriented medially in dysplastic trochlear compared with the healthy trochlear [16]. Studies had also reported that the asymmetry of the medial and lateral trochlear facet increased with the severity of trochlear dysplasia [7, 15]. One research suggested the ratio of medial and lateral trochlear facets less than 0.4 had nearly 100% sensitivity and 96% specificity in distinguishing trochlear dysplasia on MRIs [15]. Therefore, asymmetrical trochlear facets and medialized trochlear groove were also important morphological characteristics of trochlear dysplasia and may have potential impacts on the surgical treatments of osseous abnormalities, especially the preoperative planning of groove deepening trochleoplasty.

The purpose of this study was to investigate the difference of trochlear width between normal and dysplastic trochlear and to analyze whether the medialization of trochlear groove was correlated with abnormal width of trochlear facets in trochlear dysplasia. We hypothesized that the trochlear center was medialized in trochlear dysplasia due to the decreased width of the medial trochlear facet and increased width of the lateral trochlear facet.

## Materials and methods

### Patients

All procedures in this study were following protocols of the institutional review board, and the clinical data of consecutive patients who received surgical treatment due to recurrent patellar instability and trochlear dysplasia at our institutions from 2018 to 2021 were retrospectively analyzed. All patients had received one-millimeter slice CT scanning of the knee joint before surgery in the supine position and with the knee in extension and relaxed. Patients with the following conditions were excluded from this study: past surgery history of the knee joint, signs of knee osteoarthritis, skeletal immature, and incomplete CT data. The trochlear dysplasia was defined as a trochlear sulcus angle greater than 145° on axial CT scans [3]. A total of fifty patients (38 females and 12 males with an average age of 21.3 ± 6.16 years old) were included in this study. And fifty sex and age-matched patients with CT scans of the knee joint from the group of anterior cruciate ligament tear without trochlear dysplasia (sulcus angle < 145°) and patellofemoral disorders (40 females and 10 males with an average age of 22.5 ± 6.48 years old) were included as the normal group.

### Radiological measurements

**Fig. 1:**
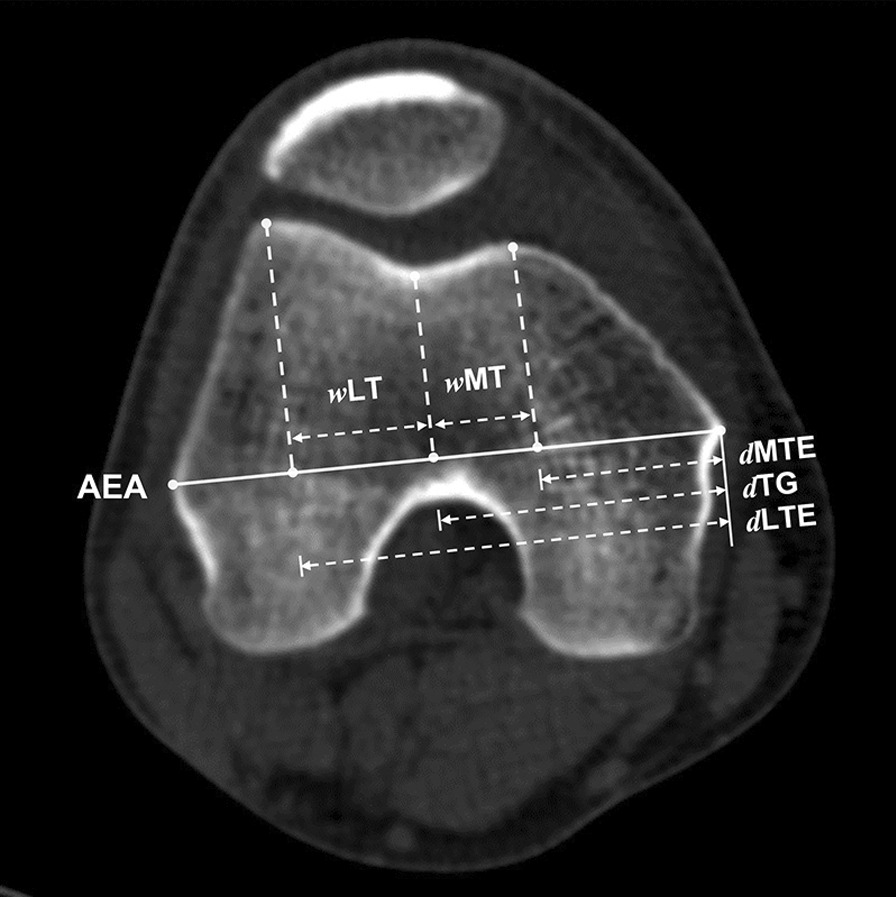
Measurements on the proximal trochlear. AEA, Anatomical epicondylar axis; *w*MT, Width of medial trochlear facet; *w*LT, Width of lateral trochlear facet; the *d*MTE, Linear distance of the medial trochlear edge; the *d*LTE, Linear distance of the lateral trochlear edge; and *d*TG, Linear distance of the trochlear groove

All parameters were measured on the transverse computed tomography at the levels when the proximal trochlear facets got full exposure. The definition of the parameters is summarized in Table 1. The anatomical epicondylar axis (AEA) connecting medial epicondyle and lateral epicondyle was chosen as the transverse width of the distal femur. The medial edge of medial trochlear facet, the center of trochlear groove, and the lateral edge of lateral trochlear facets were selected as the landmark and the linear distance to the medial epicondyle parallel to the AEA was measured, respectively. The lowest point of trochlear groove on the axial CT image was selected as the trochlear center. The trochlear facets and sulcus were obvious to be identified for the concave trochlear. For the flatted and convex trochlear, the researchers scrolled the adjacent slices to find the lowest point of the sulcus and trochlear edge. The width of medial and lateral trochlear facets was measured (Fig. 1). All the measured values were standardized with the AEA width, respectively, and shown in percentages to reduce the impacts of individual variation.

**Table 1:** Descriptions of the measurement methods

Parameters	Abbreviations	Definition
Anatomical epicondylar axis	AEA	Line connecting the prominences of the medial and lateral epicondyles on axial view
Linear distance of medial trochlear edge	*d*MTE	Linear distance from the medial epicondyle to medial trochlear edge parallel to the AEA
Linear distance of lateral trochlear edge	*d*LTE	Linear distance from the medial epicondyle to lateral trochlear edge parallel to the AEA
Linear distance of trochlear groove	*d*TG	Linear distance from the medial epicondyle to the lowest point of the proximal trochlear groove parallel to the AEA
Medial trochlear width	*w*MT	Linear width of *d*TG minus *d*MTE
Lateral trochlear width	*w*LT	Linear width of *d*LTE minus *d*TG

### Statistical analysis

The Shapiro–Wilk test was performed to test the normality of the measurements in this study. All measurements were in line with the normal distribution and present by mean and standard deviation. The statistical significance between groups was tested using two independent sample *t* test (SPSS, v24.0, IBM, Armonk, USA). The post hoc analysis of this study showed an effect size of 0.3, a power of 0.6 for the correlation analysis, and an effect size of 0.5, a power of 0.8 for the *t* test (G*Power, v.3.1, Dusseldorf, Germany) [17]. Statistical significance was set at a *P* value of less than 0.05. Two experienced surgeons repeated the measurements of randomly selected 30 cases independently with an interval of four weeks. The reliability of the measurements was evaluated using the intraclass correlation coefficient (ICC). The results of ICC indicated poor agreement when ranged from 0 to 0.2, fair from 0.21 to 0.4, moderate from 0.41 to 0.6, substantial from 0.61 to 0.8, excellent from 0.81 to 1.0. The correlation between the parameters was analyzed using Pearson’s correlation coefficient. The results of the correlation coefficient were distributed from − 1 to 1, and classified as the following: strong (0.6 to 0.8), moderate (0.4 to 0.6), or mild (0.2 to 0.4).

## Results

The reliability of the results was good (ranged from 0.89 to 0.97) (Table 2).

**Table 2:** The reliability of the results

Parameters	Interrater ICC (95% CI)	Intra-rater ICC (95% CI)
Width of anatomical epicondylar axis	0.935 (0.934 ~ 0.966)	0.977 (0.964 ~ 0.987)
Width of medial trochlear facet	0.945 (0.941 ~ 0.962)	0.946 (0.923 ~ 0.967)
Width of lateral trochlear facet	0.929 (0.933 ~ 0.965)	0.938 (0.929 ~ 0.945)
Linear distance of medial trochlear edge	0.920 (0.911 ~ 0.944)	0.933 (0.914 ~ 0.954)
Linear distance of lateral trochlear edge	0.940 (0.912 ~ 0.955)	0.956 (0.934 ~ 0.977)
Linear distance of trochlear groove	0.890 (0.875 ~ 0.934)	0.923 (0.910 ~ 0.944)

**Fig. 2:**
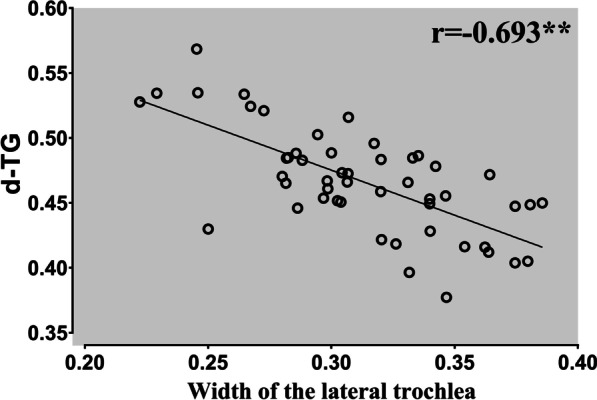
The correlation between *d*TG and the width of lateral trochlear. The *d*TG showed a significant correlation with the width of lateral trochlear, indicating that the medialization of the trochlear center may be caused by the increased width of the lateral trochlear facet. **, Correlation is significant at the 0.01 level (2-tailed)

**Fig. 3:**
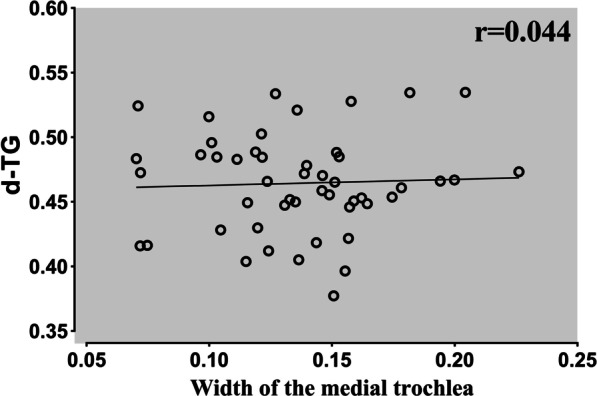
The correlation between *d*TG and the width of the medial trochlear. No significant correlation between the *d*TG and the width of the medial trochlea was found

In the TD group, there was a significant reduction in the medial trochlear width (13.9 ± 4.1% vs. 19.4 ± 2.9%, *p* < 0.001) and a significant increase in the lateral trochlear width (31.3 ± 4.0% vs. 26.9 ± 3.6%, *p* < 0.001) compared with the normal group. The *d*MTE was significantly greater in the TD group than the normal group (32.7 ± 5.1% vs. 29.6 ± 3.5%, *p* = 0.009). There was no significant difference in the *d*LTE between groups (77.9 ± 3.2% vs. 76.0 ± 5.7%, *p* = 0.068). The *d*TG was reduced in the TD group compared with the normal group (45.2 ± 4.1% vs. 49.1 ± 3.9%, *p* = 0.019). The *d*TG was significantly correlated with the lateral trochlear width (*r* value = − 0.693, *p* < 0.001) and not correlated with the medial trochlear width (*r* value = 0.044, *p* = 0.766) in trochlear dysplasia (Figs. 2 and 3). Results of the measurements are shown in Table 3.

**Table 3:** Results of the measurements

	TD group (%)	non-TD group (%)	*p* value
*d*MTE	32.7 ± 5.1	29.6 ± 3.5	0.009
*d*TG	45.2 ± 4.1	49.1 ± 3.9	0.019
*d*LTE	77.9 ± 3.2	76.0 ± 5.7	0.068
*w*MT	13.9 ± 4.1	19.4 ± 2.9	< 0.001
*w*LT	31.3 ± 4.0	26.9 ± 3.6	< 0.001
Medial–Lateral ratio	0.45 ± 0.17	0.73 ± 0.14	< 0.001

*d*MTE, Linear distance of the medial trochlear edge parallel to the AEA; *d*LTE, linear distance of the lateral trochlear edge parallel to the AEA; *d*TG, linear distance of the trochlear groove parallel to the AEA; *w*MT, medial trochlear width; *w*LT, lateral trochlear width; and Medial–Lateral ratio, the ratio of wMT and wLT.

## Discussion

The findings of this study confirmed our hypothesis that the width of medial and lateral trochlea was altered and the trochlear center was medialized in trochlear dysplasia measured by linear measurements. Compared with normal trochlear, the width of medial trochlea was significantly reduced in dysplastic trochlear, and the width of lateral trochlear was significantly increased. The distance of the medial edge of the trochlear facet relative to the medial epicondyle was increased in the TD group which indicated the narrowness of the medial trochlear facet. There was no significant difference in the distance of the lateral edge of trochlear facet between groups. Additionally, in the TD group, the medialization of the proximal trochlear groove was correlated with the increase in the lateral trochlear width.

The medial hypoplasia of trochlear facets featured with a flattened medial slope was recognized as one of the most distinctive features of trochlear dysplasia. Researchers had quantified the morphology of distal femur condyle and found that the decreased medial condylar height and width related to the hypoplastic medial trochlear were the major deformities of trochlear dysplasia [13]. The reduced medial trochlear found by our study was consistent with the previous findings. In addition, the increased distance between the medial epicondyle and medial trochlear edge proposed that the hypoplasia of medial trochlear may be started from the medial edge of the trochlear facet.

In individuals, geometry of the dysplastic trochlear was complicated to be evaluated quantitatively [18]. The anterior condylar height and trochlear groove depth were commonly used measurements to quantify the morphology of trochlear dysplasia [7]. Ferlic et al. had reported excellent reliability of the linear measurements in quantifying the dysplastic trochlear [12]. Some researchers had reported that a trochlear depth of less than 3 mm and a medial-to-lateral facet ratio of less than 40% were considered pathological in trochlear dysplasia [15]. In this study, the tangent distance of trochlear edge and trochlear width was assessed relative to the anatomical trans-epicondylar axis. Compared with measuring the length of inclined trochlear slope, these measurements with the reference of medial epicondyle may directly reflect the relative orientation of the trochlear on the distal femoral condyle.

A tibial tubercle-trochlear groove distance of greater than 20 mm was commonly used as a surgical indicator for the medialization tibial tubercle osteotomy. Some people reported that increased tibial tuberosity-trochlear groove (TT-TG) distance was due to the medialization of trochlear groove by measuring the distance between the trochlear groove and the tibial landmarks [16]. Some literature supported that the TT-TG distance was greater in recurrent patellar dislocation, but the extent of tibial tubercle lateralization was not substantial [19]. The various reference points measuring the trochlear groove raised a problem that whether medialization of trochlear groove was caused by the trochlear dysplasia or by the torsion of lower limbs. Studies had reported that degrees of femoral torsion or knee torsion also influenced the measurement of tibial tuberosity-trochlear groove distance and patellofemoral congruence [3, 20, 21]. Tibial tubercle osteotomy was to transfer the tibia tubercle medially. The deepening trochleoplasty needed to remodel the trochlear groove and oriented the groove laterally. The primary deformities may have crucial influences on whether to perform tibial tubercle osteotomy or trochleoplasty accompanied by medial patellofemoral reconstruction. By using the reference points on the distal femoral condyle, the findings of our study demonstrated the medialization of trochlear groove in trochlear dysplasia besides the torsional factors.

Morphologically, trochlear dysplasia was featured by irregular surface from the proximal trochlear [3, 22]. In this study, the *d*TG was decreased relative to the medial epicondyle indicated that the medialization of the dysplastic trochlear groove. And the lateral trochlear width was increased in the dysplastic trochlear compared with the normal trochlear. Besides these morphological findings, the present study found a significant correlation between the lateral trochlear width and medialization of the trochlear center. Besides, there was no significant change in the position of lateral trochlear edge between groups, indicating that the position of lateral trochlear facets was not lateralized. Hence, it was speculated that the medial expansion of lateral trochlear was the reason for the medialization of the proximal trochlear groove in trochlear dysplasia.

The etiology of recurrent patellar instability was multifactorial [22–24]. Trochlear dysplasia was a major risk factor for patellar instability. The development of trochlear dysplasia was still controversial. Studies had reported that minor changes in trochlear morphology during skeletal growth and indicated that trochlear dysplasia was genetically determined [22, 25]. According to the researchers, the dysplastic trochlear could be surgically induced using the animal model of patellar subluxation [26, 27]. The trochlear bump and distance between the proximal trochlear and distal femoral physis increased with age which indicated the role of acquired factors on trochlear development [28]. The skeletal growth could be strengthened by applied stress according to Wolff's law [29]. The increased lateral trochlear in trochlear dysplasia might be associated with increased sliding pressure caused by the lateralized patellar tracking. Hence, the findings of this study also implied the influence of mechanical status on the development of trochlear dysplasia.

This study had some limitations. Firstly, this study was a retrospective study. The patients and their matched controls in this study were selected from consecutive cases admitted to our hospital in a limited period. To diminish potential selection bias, additional independent studies were required to validate the findings of this study. Secondly, this study evaluated the trochlear dysplasia on the proximal part where the patella engaged with the trochlear sulcus at the beginning of knee flexion. Biedert et al. had reported the characteristics of trochlear dysplasia by separating the trochlear into the proximal and distal parts [30]. Further researches on the characteristics of the entire trochlear were needed to reveal the pathological changes of the trochlear. Lastly, although this study had detected significant results, based on the average femoral condyle width of 70 mm, medialization of the trochlear groove in these patients was only approximately 3 mm compared with that of the normal trochlear. The results cannot fully explain the increased TT-TG distance in patients with recurrent patellar instability, so a detailed evaluation is needed before surgical treatments.

In conclusion, this study demonstrated that dysplasia of trochlear morphology was related to the reduction of medial trochlear width and increase in lateral trochlear width. The medialization of trochlear groove was significantly correlated with the increased width of lateral trochlea.

## Data Availability

All of the data and materials are available.

## Abbreviations

dMTE: Medial edge of medial trochlear facet
dTG: Trochlear groove
dLTE: Lateral edge of lateral trochlear facets
AEA: Anatomical epicondylar axis
ICC: Intraclass correlation coefficient
TT-TG: Tibial tuberosity-trochlear groove
